# Prevalence and Geographic Distribution of Self-Reported Chronic Kidney Disease and Potential Risk Factors in Central America

**DOI:** 10.3390/ijerph20021308

**Published:** 2023-01-11

**Authors:** Erika Figueroa-Solis, David Gimeno Ruiz de Porras, Marianela Rojas-Garbanzo, Lawrence Whitehead, Kai Zhang, George L. Delclos

**Affiliations:** 1 Department of Epidemiology, Human Genetics and Environmental Sciences, The University of Texas Health Science Center at Houston (UTHealth) School of Public Health, Houston, TX 77030, USA; 2 Southwest Center for Occupational and Environmental Health, Department of Epidemiology, Human Genetics and Environmental Sciences, The University of Texas Health Science Center at Houston (UTHealth) School of Public Health in San Antonio, San Antonio, TX 78229, USA; 3 Instituto Regional de Estudios en Sustancias Tóxicas (IRET), National University of Costa Rica, Heredia 40101, Costa Rica; 4 Southwest Center for Occupational and Environmental Health, Department of Epidemiology, Human Genetics and Environmental Sciences, The University of Texas Health Science Center at Houston (UTHealth) School of Public Health, Houston, TX 77030, USA; 5 Department of Environmental Health Sciences, School of Public Health, University at Albany, State University of New York, Albany, NY 12144, USA

**Keywords:** chronic kidney disease (CKD) of undetermined origin, workers, industry sector, temperature

## Abstract

Background: Cases for chronic kidney disease of unknown etiology (CKDu) are increasing in specific disease hotspots located in rural agricultural communities over Central America. The goal of the study was to estimate the prevalence and geographic distribution of self-reported work-related CKD and associated risk factors for CKDu by industry sector in Central America. Methods: We calculated the prevalence and distribution of self-reported CKD, work-related CKD, and suspected CKDu risk factors among the 9032 workers in the Second Central American Survey of Working Conditions and Health (II ECCTS, 2018). We mapped the distribution of suspected CKDu risk factors to work-related CKDu and weather conditions using average annual temperatures. Results: The primary and secondary industry sectors showed the highest proportion of males, suspected CKDu risk factors, and work-related CKD. Age (30–49 years: OR = 2.38, 95% CI 1.03–5.51), ethnicity (mestizo: OR, 7.44, 95% CI: 2.14–25.82), and exposure to high physical work demands (OR = 2.45, 95% CI: 1.18–5.09) were associated with work-related CKD. The majority of work-related CKD were reported in the western parts of Honduras and Nicaragua, in hot temperature regions, and overlapped with those areas with a high density of CKDu risk factors. Finally, some areas clustered CKDu risk factors without any work-related CKD points, mainly in the western part of Guatemala. Conclusion: Our findings supplement prior CKDu findings regarding a high prevalence of work-related CKD among 30- to 49-year-old mestizo males in the primary and secondary sectors, in hot temperature areas, in the central and western region, and overlapping with persons reporting two or more CKDu risk factors. Moreover, several geographic areas with CKDu risk factor clusters had no reported work-related CKD. These areas represent new industries and sectors to be monitored for possible future increases of CKDu cases.

## 1. Introduction

Chronic impairment of kidney function not associated with known risk factors, or a specific histological diagnosis, has been termed “chronic kidney disease (CKD) of undetermined origin” (CKDu) [1,2,3,4]. This type of chronic kidney disease is not associated with established risk factors, such as hypertension or diabetes [5,6]. In Central America, CKDu is hypothesized to be associated with occupational and environmental exposures mainly affecting young men working in lowland agricultural settings, most notably sugarcane harvesters [7,8]. It is more prominent in men who have worked in such settings for two or more seasons, are between 20 and 50 years old, are asymptomatic, and have normal or only slightly elevated blood pressure and normal blood glucose levels [9]. By 2012, an estimated 20,000 deaths were attributed to CKDu [5]. Cases for CKDu are increasing and presenting in specific disease hotspots, most often located in rural agricultural communities [10]. This disease hotspot concept creates a geographical construct that can be identified, visualized, and explored using geographic information systems (GIS) and spatial analysis methods [11].

Other industries that share similar risk factors could conceivably also harbor CKDu [12,13,14,15,16]. There is a great need for standardized tools to estimate potential kidney disease prevalence. For exploratory or hypothesis-generating purposes, data derived from national surveys of working conditions and health could serve to identify clusters of self-reported renal disease and/or its risk factors. Any such clusters could then warrant further investigation. The First Central American Survey of Working Conditions and Health (I ECCTS by its Spanish acronym) was conducted in 2011 and included its six Spanish-speaking countries (from North to South: Guatemala, Honduras, El Salvador, Nicaragua, Costa Rica, and Panama), where over 12,000 workers were interviewed in their homes [17]. Neither questions specifically directed at suspected risk factors for CKDu (i.e., exposure to heat, hydration status, physical exertion, nonsteroidal anti-inflammatory drugs (NSAIDs) use, etc.) nor a specific question on pre-existing kidney disease was included. A second round of the survey, the II ECCTS, was administered in 2018 in the same six countries to over 9000 workers. As was the case in the I ECCTS, the study population consisted of large, nationally representative samples of the working population. This newer survey, however, featured a supplemental “Heat/Kidney Disease” module that included self-reported items specifically targeting the suspected CKDu risk factors, as well as a question on kidney disease.

Linking survey data to their geographic location and then location-based information such as weather conditions could add an element of validation to self-report as well as supplemental information on the identification and distribution of possible clusters of CKDu and its risk factors. Recent advances in the GIS field have allowed agriculture sectors to estimate water availability and drought frequency that affect both human and environmental systems [18,19]. Working under hot, dry, and humid conditions, involving tasks characterized by high physical demands, can cause workers to become dehydrated and increase their chance of developing heat stress if fluids and minerals are not adequately replenished [20]. Repeated exposure to this type of environment can affect renal function, possibly through repeated episodes of acute kidney injury (AKI) that can potentially lead to chronic disease [21].

Using data from the II ECCTS and GIS linkages, we estimated the prevalence and geographic distribution of CKD, work-related CKD, and suspected CKDu risk factors. We also mapped the geographic variations of temperature patterns in relation to CKDu in Central America.

## 2. Materials and Methods

### 2.1. Sample Recruitment and Data Collection

The II ECCTS survey was administered to a nationally representative sample of at least 1500 workers per country (9032 overall, from 1500 in Nicaragua to 1510 in Guatemala) and included both sexes, formal and informal workers, and both urban and rural settings [22]. The sampling frame has been previously described [17,22]. Results were weighted by country, age, sex, and industry sector: primary (mainly agricultural), secondary (mainly manufacturing and construction), tertiary (mainly services). Data collection was performed between February and June 2018. Based on the I ECCTS questionnaire [17], the II ECCTS included standard questions used in previous international surveys and sections on demographics, employment conditions, labor rights, working conditions, and health and well-being indicators. The II ECCTS had additional newly developed items on CKDu and its suspected risk factors [22].

### 2.2. Measures

The outcome of CKD was measured by a Yes/No response to the question: “In the last month, have you experienced the following: Chronic kidney disease (kidneys)?” If answered yes, participants were asked whether or not they thought their disease was work-related (i.e., self-reported work-related CKD). Suspected work risk factors included the following: physical work demands, measured by asking about the physical effort or the work intensity carried out at work using a visual aid and 5-point Likert scale: very easy, easy, strong, very strong, and so strong that I have to take breaks; exposure to heat (high temperatures at the workplace that made you feel uncomfortable) and humidity (general level humidity at the workplace), both of them measured using a 4-point Likert scale (frequently, sometimes, rarely, never); water intake measured using a 6-point Likert scale (every 30 min or with more frequency, every hour, every hour and a half, every two hours, every four hours, and I did not drink water at work); being highly thirsty at the jobsite measured using a 3-point Likert scale (not thirsty, somewhat thirsty, and highly thirsty); use or direct contact with agrochemicals over the last 12 months measured using a 4-point Likert scale (frequently, sometimes, rarely, never); and use of analgesics over the last week was an open-ended response.

Information on the following sociodemographic variables was also collected: (1) sex: male, female; (2) age group: 18–29, 30–49, 50–64, 65+ years; (3) ethnicity: indigenous, mestizo, white, black, mulatto; (4) education: no schooling, elementary, middle school, university; (3) average monthly income: no more than $200, $201 to $300, $301 to $500, $501 to $1000, or more than $1000; (4) employment time: less than one year, up to five years, six to 10 years, 11 to 15 years, 16 years and above; (5) zone: urban, rural; (6) sector: primary (agriculture, fishing, mining), secondary (construction, manufacturing), and tertiary (services).

### 2.3. Statistical Analysis

We calculated the weighted prevalence of the sociodemographic variables and of self-reported CKD (yes or no), self-attributed work-related CKD (yes or no), and each of the seven suspected CKDu risk factors: high physical job demands (responses “strong,” “very strong,” and “so strong I have to take breaks” coded as “Yes”); exposure to heat/high temperatures (“frequently” coded as “Yes”); exposure to humidity (“very humid and muggy” coded as “Yes”); no water intake (“I don’t drink water at work” coded as “Yes”); high thirst (“I’m very thirsty” coded as “Yes”); use of agrochemicals (“frequently” coded as “Yes”); and, use of analgesics (using one or more analgesic codes as “Yes”). To estimate the association of CKD and work-related CKD with other variables, we used logistic regression models following Hosmer and Lemeshow’s model-building strategy [23]. Hosmer-Lemeshow goodness-of-fit tests were run to determine the models fit, which is indicated by a p-value greater than 0.5 [24]. All statistical analyses were performed using Stata v.16 [25].

We generated static GIS weather maps to examine links between weather patterns, work-related CKD, suspected CKDu risk factors, and geographic buffer zone location of the workplace reported by the participants, using ArcGIS 10.x ^®^ [26]. We first linked the de-identified survey data to GIS weather maps by GPS coordinates to characterize the climate patterns and the geographic distribution of CKD and CKDu risk factors. Average annual temperature readings were collected from the National Oceanic and Atmospheric Administration (NOAA) and Time and Date under verified weather data websites [27,28]. Interpolation was completed using the weather station readings to estimate the average annual temperature of participants’ outdoor work environments. To estimate workplace locations, location coordinates were measured and compared to the survey questions asking for means of transportation, how long it takes to arrive at the job, and the type of place where the work is located (e.g., at a house, building, or country) in order to calculate distance traveled. A buffer zone of 10 miles was created from the original location coordinate to the estimated distance of the workplace location. Suspected CKDu risk factors (physical demands, heat, and humidity on the job) were selected [8,10], and a map was created for answers coded as “yes” to any two of these three CKDu risk factors.

## 3. Results

In the weighted sample of workers in the total of Central America, there were more men than women (61% vs. 39%) (Table 1). The proportion of males was much larger in the primary sector than in the two other sectors. Many participants were in the 30- to 49-year age group, had a middle school education, and earned less than $200 a month. The most frequently reported CKDu risk factors were high physical work demands (49%) and the use of analgesics (87.2%). Overall, 4.4% of the participants reported a history of CKD (*n* = 370); 38% (*n* = 141) of these were further reported as work-related CKD. When comparing across industry sectors, workers in the primary sector reported higher percentages for middle school education, earning less than $200 a month, and five CKDu risk factors (high physical work demands, exposure to heat and humidity at work, high use of agrochemicals, and having high thirst). The majority of workers in the secondary and tertiary sectors reported a university education, earning at least $500 a month, and one CKDu risk factor (no water intake). Close to half of the workers across all industry sectors had been employed at least five years in their current job.

The CKD and work-related CKD groups had an elevated prevalence of high physical work demands (63% and 75%, respectively) and use of analgesics (78% and 71%, respectively) (Table 2). Workers in the primary and secondary sectors who reported CKD and work-related CKD had higher percentages of high physical work demands, exposure to heat and humidity at work, increased use of agrochemicals, and high thirst than those in the tertiary sector. For both CKD and work-related CKD, high analgesic use and no water intake at work were higher in the tertiary sector.

**Figure 1:**
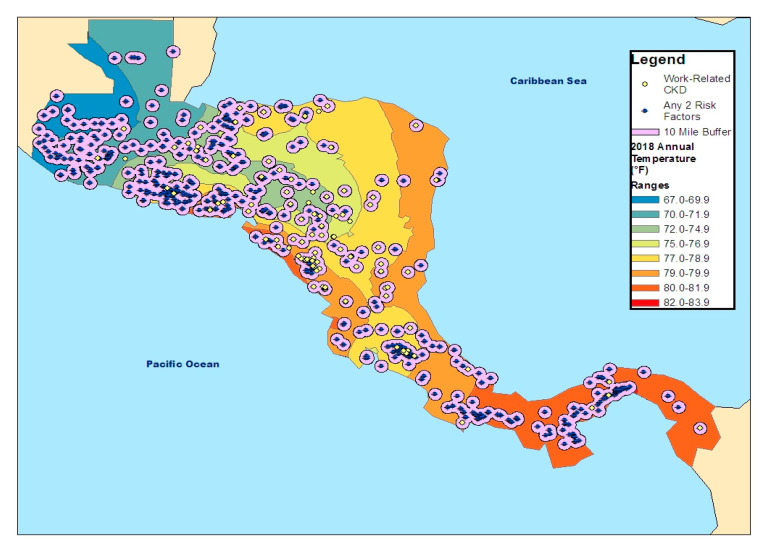
Geographic distributions of work-related CKD cases and CKDu risk factors (high temperature, high humidity, high physical demands).

The weather map and its linkages to work-related CKDu and persons with two or more CKDu risk factors for Central America are shown in Figure 1. The majority of work-related CKD GPS coordinates were located in the western parts of Honduras and Nicaragua, with scattered cases in Guatemala, Costa Rica, and the Pacific coast of Panama. Risk factors for CKDu (exposure to heat and humidity at work and high physical work demands) were more commonly (~54%) reported in Guatemala, El Salvador, and Honduras, and along the coastal areas. Most work-related CKDu overlapped with areas with a high density of CKDu risk factors (~65%). However, there were a few work-related CKD coordinates that did not have this overlap (~32%), primarily in central Nicaragua and isolated instances along its Atlantic coast. Conversely, there were areas that clustered CKDu risk factors without any work-related CKD points (~15%), chiefly in the western part of Guatemala.

Annual average temperatures in 2018 were cooler in the northern parts of Central America (67.0° F–74.9° F), while the highest annual average temperatures (79.0° F–83.9° F) were along the coastal regions of Nicaragua and Panama. Most work-related CKD coordinates were located in warm temperature regions (75.0° F and above). The CKDu risk factors were present across all temperature ranges but more so in relatively cool areas (67.0° F–74.9° F) or hot areas (70.0° F–81.9° F).

## 4. Discussion

In this large-scale, population-based epidemiologic study using nationally representative samples of workers, we identified areas of suspected work-related CKDu and its risk factors in Central America. Young men without the common CKD risk factors or underlying conditions, such as diabetes or hypertension, but who work in physically demanding jobs under conditions of high heat and humidity are at high risk of CKDu [1,9]. Our findings supplement the prior literature demonstrating a high prevalence of self-attributed work-related CKD among 30- to 49-year-old males who worked in the primary and secondary sectors and had physically demanding jobs. Our findings are novel in our use of GIS, showing a geographic concentration of self-reported work-related CKD in the central to the western region of Central America, which is associated with hot temperatures and overlaps with a higher frequency of people reporting two or more CKDu risk factors. These areas in Nicaragua, El Salvador, and Costa Rica are well-described in the literature as focal areas of CKDu [10,12,14,29,30,31,32,33]. However, there are less well-known areas, such as the central part of the Central America region, particularly Honduras, where cases are less often described and that our findings suggest should be further studied. In addition, we also found several areas with a concentration of CKDu risk factors but with no reported work-related CKD in central Guatemala as well as the coastal areas of Panama and southern Costa Rica. These areas represent an opportunity for follow-up with studies that include measurement of renal function to identify possibly as yet undeclared clusters of CKDu cases.

We observed that work-related CKD was associated with sociodemographic variables, mainly sex, age, and ethnicity. This is consistent with the target population: middle-aged men between 30 and 50 years old [8,10,29,34]. Although the mestizo ethnicity was also associated with work-related CKD, previous studies have yet to find CKD-risk to be ethnicity-specific to the mestizo population [35,36]. The term “mestizo” is used to identify populations who have an extensive admixture of Native American, European, and African ancestry [37]. Two ethnicity-specific genes have been associated with end-stage renal disease of unknown etiology in other populations: apolipoprotein L1 (APOL1) gene in African Americans, and Glutathione S-transferase Mu 1 (GSTM1) null phenotype in the Mexican population [38,39,40]. This evidence has led the authors of these studies to suggest a possible association of genetic factors or variants with the mestizo population. However, no such studies have addressed this to any great extent in the Central American mestizo population. Thus, further studies are needed to better understand the role of ethnicity in CKDu [30,35,41].

High humidity, no water intake, and high thirst were associated with those participants that reported CKD, while high physical demands were associated with work-related CKD. All four of these occupational risk factors are well documented as having a role in CKDu [42,43,44]. In the majority of research done in Central America, age, male sex, low altitude, high ambient temperatures, and dietary history have been positively associated with CKDu [13,30]. We found high humidity and no water intake to be associated with CKD. In a recent detailed review, Wesseling and colleagues concluded working-age men exposed to heat, humidity, intensive labor, and without proper hydration, are more susceptible to developing CKDu in Central America [8]. The use of analgesics was not found to be associated with either CKD group. This finding is in accordance with the lack of evidence from epidemiologic studies [30,36,45]. Although exposure to agrochemicals was not found to be a risk factor in our study, some studies have found agrochemical use to possibly have a role in the development of CKDu due to high mortality rates in both men and women [46,47]. However, findings related to the role of agrochemicals in CKDu have been inconsistent, with most not finding evidence of a link [30]. Finally, the strong relationship we found between CKD and established risk factors for CKDu may also indicate that some of these CKD cases are occurring in persons who have yet to recognize its work-relatedness.

Creating the weather map allowed us to visualize where self-reported work-related CKD cases are located in relation to CKDu risk factors in Central America. Most of the work-related CKD was located in the central to western parts of the region, with very few cases along the Caribbean coast. Several studies of CKDu conducted in Guatemala have involved the Escuintla region, located in the country’s coastal lowland region, where sugarcane harvest is common, along with conditions of high humidity, heat, and heavy physical demands [48,49,50,51]. Similar conditions are found along the coastal areas of El Salvador, Nicaragua and the Guanacaste region of Costa Rica [31,33,52]. However, we also observed clusters of CKDu risk factors together with work-related CKD in geographic areas that are less often described; specifically, western Honduras and the Pacific coasts of lower Costa Rica and Panama. In Honduras, the northern coastal region along the Caribbean and the surrounding land by the Gulf of Fonseca is arable land used primarily for the cultivation of crops such as bananas, coffee, corn, tobacco, and cotton [53]. In the lower Costa Rican coast, there is a large palm industry, with harvest being done under very humid environments since workers perform their jobs under the low-laying palm tree canopy [54,55]. In Panama, the coastal area has large fishing and banana harvest industries that are essential to their economy, typically performed under conditions of high physical demand and humid conditions [56]. We also identified areas that clustered CKDu risk factors but in the absence of any work-related CKD. This occurred mainly in the western part of Guatemala, coastal areas of Panama, and southern Costa Rica. Our findings may be pointing to industry sectors, other than agricultural, harboring workers susceptible to the development of CKDu. Targeted follow-up studies of workers in these areas, with surveys and measurement of renal function, would serve to identify the degree to which CKDu is an issue there.

Lastly, we also encountered a few work-related CKD reports that did not overlap with CKDu risk factors, primarily in central Nicaragua. Most CKDu studies conducted in Nicaragua have reported CKD in working-age men [12,57,58]. Agricultural regions for coffee, cotton, and sugarcane in Nicaragua are consistent with our work-related CKD cluster findings. However, the absence of overlying CKDu risk factors in these few cases could either be spurious or suggest that some work-related CKDu could be associated with different occupational risk factors, or not be work-related given the limitations of self-report. A recent study has also suggested that the cultivation of coffee may not be associated with CKDu risk factors [6].

A major strength of this study is that it is based on a multi-country survey with a large sample size, representative of the working population in Central America, which increases the generalizability of findings. The inclusion of both informal and formal sector workers also makes it possible to search for CKDu in populations that often escape official registries despite being more vulnerable and likely exposed to more hazardous working conditions [17,59]. Because of the study population size and sampling frame, we were also able to expand our study of renal disease and its risk factors to different industry sectors, going beyond agriculture. In fact, the prevalence of self-reported CKD and work-related CKDu in the secondary and tertiary sectors was not trivial. Recent studies of nonagricultural workers who may share similar work exposures such as high heat and physical demands have identified other occupations, such as construction workers or brickmakers [14,29], or groups of vulnerable workers such as Hispanic outdoor workers [60]. Linkages of our GIS weather maps to survey responses added more objectivity to self-reports of exposure to high temperatures and humidity. A recent large-scale ecological study in Mesoamerica (Mexico and Central America) modeled the spatial distribution of CKD burden ratio and hot cane-cultivating municipalities in weather conditions below 30 °C in agricultural working-age men [6]. The areas with high CKD burden and high cane density in areas identified in that study overlap with the areas with large clusters of CKDu risk factors we have identified. Though more studies are necessary, this finding further heightens the probable association between work intensity, temperature, and CKDu.

There are also some limitations to consider when interpreting our findings. First, all data were self-reported, making it susceptible to recall bias. Participants without a clinical diagnosis of renal disease are less likely to know or suspect they have CKD, leading to underreporting of disease. On the other hand, participants with diagnosed CKD could inflate causal attribution since they may have a higher tendency to attribute their health condition to their work [61]. Previous studies have confirmed humidity is a contributing risk factor for CKDu [8,43]. It is possible that using the heat index as a measure of heat exposure would have yielded a stronger association between heat exposure as a possible CKDu risk factor for work-related CKD as it reflects the combined effect of temperature and relative humidity. However, heat index data were not available in the 2018 weather station readings, limiting the ways in which we could examine the effects of temperature on self-reported renal disease [62]. It is also important to note that the annual temperatures for heat demonstrated in the map may not affect all workers continuously year-round. For example, sugarcane workers only work during harvesting seasons, which can last for six months. Once the harvesting season is over, agricultural workers pursue work opportunities in jobs that may or may not be outdoors. Thus, constant year-round exposure to heat and humidity in the agriculture sector cannot be measured accurately. Nonetheless, continuous day-to-night exposure to heat among other suspected CKDu risk factors for a period of 6 months can accelerate the symptoms for AKI, which can later progress to CKDu [63,64,65,66].

## 5. Conclusions

Our study results merely provide a bird’s eye view of population-based prevalence of chronic kidney disease and its associations with putative occupational risk factors. Findings from our GIS analysis are more aligned with an ecological design, and thus serve more as hypothesis-generating, identifying opportunities for more targeted and more rigorously designed studies. Next steps in this direction could include studies that couple survey results with objective, blood-based measurement of renal function, such as obtaining baseline levels of estimated glomerular filtration rates (eGFR and implementing the Disadvantaged Populations eGFR Epidemiology (DEGREE) [4]. A small pilot study conducted in Houston, TX in Hispanic outdoor workers tested this coupled approach [60]. There is also a need for better weather data collection from existing Central American weather stations. Finally, expanding this epidemiologic approach to other Hispanic worker populations, including those in the U.S. subject to similar meteorological conditions, occupations, and physical demands, and across all industry sectors, could lead to the detection of new clusters of CKDu [67]. Detection of new disease is the first step in identifying opportunities for the design of preventive interventions aimed at reducing the burden of CKDu in these workers.

## Figures and Tables

**Table 1:** Demographics of participants in II ECCTS (*n* = 9032) for Central America.

	Primary Sector ^+^ N (%) *	Secondary Sector ^+^ N (%) *	Tertiary Sector ^+^ N (%) *	Total
Sex Male Female	812 (84.4%) 146 (15.6%)	1015 (58.9%) 605 (41.1%)	2735 (41.7%) 3719 (58.3%)	4562 (61.3%) 4470 (38.7%)
Age (years) 18–29 30–49 50–64 65+	200 (27.9%) 395 (37.5%) 245 (23.1%) 118 (11.6%)	403 (38.5%) 766 (39.6%) 350 (16.4%) 101 (5.5%)	1831 (42.5%) 2871 (36.9%) 1350 (15.3%) 402 (5.3%)	2434 (36.4%) 4032 (38.0%) 1945 (18.2%) 621 (7.4%)
Ethnicity Indigenous Mestizo White Black Mulatto Other/ Do not know/Refused	198 (30.3%) 506 (48.2%) 104 (10.5%) 24 (1.5%) 25 (1.8%) 101 (7.7%)	202 (25.4%) 806 (50.2%) 286 (11.0%) 63 (1.2%) 41 (1.1%) 222 (11.2%)	639 (21.0%) 3347 (53.3%) 1348 (12.4%) 270 (1.7%) 226 (1.4%) 624 (10.2%)	1039 (25.5%) 4659 (50.6%) 1738 (11.3%) 357 (1.4%) 292 (1.4%) 947 (9.7%)
Education (years of schooling) No schooling Elementary (Grades 1–6) Middle School (Grades 7–12) University (years 1–6) Do not know/Refused	37 (3.8%) 175 (18.1%) 562 (56.1%) 184 (22.0%) 0 (0.0%)	147 (9.1%) 75 (5.9%) 641 (41.0%) 756 (44.0%) 1 (0.0%)	1391 (18.8%) 249 (4.5%) 1868 (30.1%) 2942 (46.5%) 4 (0.1%)	1575 (10.7%) 499 (9.4%) 3071 (42.2%) 3882 (37.7%) 5 (0.0%)
Household Monthly Income No more than $200 $201 to $300 $301 to 500 $501 to $1000 More than $1000 Do not know/Refused	496 (55.8%) 187 (18.6%) 130 (16.1%) 60 (3.9%) 23 (1.4%) 62 (4.2%)	396 (32.3%) 355 (27.2%) 405 (25.2%) 288 (9.8%) 92 (2.5%) 84 (2.9%)	1608 (33.5%) 1357 (25.6%) 1606 (24.4%) 1064 (9.4%) 386 (2.5%) 433 (3.6%)	2500 (40.3%) 1899 (24.2%) 2141 (22.0%) 1412 (7.8%) 501 (2.2%) 579 (3.6%)
Length of Employment Less than 1 year Up to 5 Years 6 to 10 years 11 to 15 years 16 years and above Do not know/Refused	90 (7.6%) 244 (31.4%) 144 (14.4%) 108 (13.0%) 360 (32.7%) 12 (0.9%)	198 (11.7%) 617 (44.6%) 289 (16.5%) 138 (7.6%) 364 (18.9%) 14 (0.7%)	919 (14.5%) 2947 (51.4%) 1054 (14.7%) 515 (6.7%) 937 (11.4%) 82 (1.3%)	1207 (11.3%) 3808 (42.6%) 1487 (15.2%) 761 (9.0%) 1661 (20.8%) 108 (1.0%)
Zone Urban Rural	194 (21.0%) 764 (79.0%)	1084 (64.2%) 536 (35.8%)	4698 (71.8%) 1756 (28.2%)	5976 (52.8%) 3056 (47.2%)
CKDu Risk Factors High physical demands of work Exposure to heat Exposure to humidity High thirst No water intake Use of analgesics High exposure to agrochemicals	659 (66.3%) 274 (30.1%) 66 (4.8%) 449 (39.8%) 19 (2.1%) 785 (86.6%) 488 (50.5%)	891 (49.9%) 398 (22.1%) 61 (2.1%) 569 (31.1%) 56 (2.9%) 1315 (88.3%) 98 (3.9%)	2243 (31.6%) 1106 (16.3%) 163 (1.3%) 1728 (22.5%) 298 (4.5%) 5037 (86.6%) 363 (3.5%)	3793 (49.0%) 1778 (22.7%) 290 (2.7%) 2746(31.0%) 373 (3.2%) 7137 (87.2%) 949 (18.9%)
CKD Responded Yes Believe CKD to be work-related ^^^	64 (5.4%) 35 (41.4%)	60 (4.5%) 27 (41.2%)	246 (3.5%) 79 (27.5%)	370 (4.4%) 141 (37.5%)

* Weighted Percentage. ^+^ Industry sector: primary (mainly agricultural), secondary (mainly manufacturing and construction), tertiary (mainly services). ^^^ Percentage is out of the total of self-reported CKD.

**Table 2:** CKD risk factors by industry sectors between CKD and work-related CKD for Central America.

	CKD (*n* = 370)				Work-Related CKD (*n* = 141)			
Sectors	Primary Sector ^+^ N (%) *	Secondary Sector ^+^ N (%) *	Tertiary Sector ^+^ N (%) *	Total	Primary Sector ^+^ N (%) *	Secondary Sector ^+^ N (%) *	Tertiary Sector ^+^ N (%) *	Total
CKD Risk Factors High physical demands of work Exposure to heat Exposure to humidity High thirst No water intake Use of analgesics High exposure to agrochemicals	47 (75.0%) 22 (43.4%) 7 (7.2%) 38 (58.4%) 3 (2.0%) 43 (74.9%) 29 (50.7%)	35 (61.7%) 16 (27.0%) 3 (4.6%) 23 (43.5%) 5 (5.2%) 43 (78.1%) 2 (3.9%)	121 (45.3%) 56 (18.1%) 7 (2.1%) 92 (36.7%) 22 (8.1%) 190 (82.8%) 19 (7.0%)	203 (62.6%) 94 (31.1%) 17 (5.0%) 153 (47.5%) 30 (4.7%) 276 (78.1%) 50 (23.2%)	30 (90.4%) 13 (35.4%) 4 (9.3%) 22 (66.1%) 1 (2.3%) 23 (70.5%) 20 (67.0%)	17 (69.1%) 11 (42.9%) 2 (6.2%) 11 (44.9%) 1 (0.5%) 16 (65.0%) 1 (4.4%)	47 (53.2%) 21 (22.5%) 5 (5.9%) 33 (38.7%) 6 (6.4%) 61 (84.3%) 5 (4.6%)	94 (74.8%) 45 (35.7%) 11 (7.4%) 66 (52.5%) 8 (2.5%) 100 (71.2%) 26 (30.9%)

* Weighted Percentage. ^+^ Economic sector: primary (agricultural), secondary (manufacturing and construction), tertiary (services). Bivariate logistic regression analysis identified the following variables for selection into the multivariate analysis (*p* < 0.20): CKD: age, education, monthly income, length of employment, zone, and sector of the economy, high physical work demands, exposure to heat and humidity at work, high thirst, no water intake, use of analgesics; work-related CKD: sex, age, ethnicity, monthly income, economic sector, high physical work demands, exposure to humidity at work, no water intake (Table 3). Six CKDu risk factors (except high use of analgesics) were included in the multivariate analysis for both CKD and work-related CKD models for consistency across both dependent variables. Exposure to high humidity (OR, 2.08, 95% CI 1.07 to 4.06), no water intake (OR, 1.93, 95% CI 1.05 to 3.57), and high thirst (OR, 1.89, 95% CI 1.25 to 2.86) were associated with CKD. The 30- to 49-year age group (OR, 2.38, 95% CI 1.03 to 5.51), ethnicity (mestizo: OR, 7.44, 95% CI 2.14 to 25.82), and exposure to high physical work demands (OR, 2.45, 95% CI 1.18 to 5.09) were associated with work-related CKD. Goodness-of-fit tests for both fully-adjusted models showed a good fit (*p* > 0.2).

**Table 3:** Bivariate and multivariate logistic regression analysis of associations between CKD/work-related CKD and risk factors.

	CKD (*n* = 370)		Work-Related CKD (*n* = 141)	
Variables	OR [95% CI] Crude	OR [95% CI] Adjusted	OR [95% CI] Crude	OR [95% CI] Adjusted
Sex Female Men	Ref 1.00 [0.69–1.43]	Ref --	Ref 2.39 [1.22–4.66]	Ref 1.83 [0.88–3.81]
Age (years) 18–29 30–49 50–64 65+	Ref 1.17 [0.73–1.88] 1.71 [1.01–2.91] 1.45 [0.69–3.04]	Ref. 1.17 [0.69–1.98] 2.08 [1.23–3.52] 1.74 [0.80–3.78]	Ref 1.97 [0.82–4.74] 1.47 [0.55–3.93] 0.77 [0.23–2.65]	Ref 2.38 [1.03–5.51] 0.93 [0.31–2.82] 0.71 [0.20–2.52]
Ethnicity Indigenous Mestizo White Black Mulatto Other	Ref 1.10 [0.64–1.88] 0.85 [0.39–1.84] 0.79 [0.31–2.04] 0.51 [0.15–1.71] 0.64 [0.30–1.35]	Ref -- -- -- -- --	Ref 4.17 [1.26–13.79] 1.41 [0.30–6.59] 2.76 [0.41–18.44] 2.51 [0.26–24.02] 2.02 [0.43–9.50]	Ref 7.44 [2.14–25.82] 2.24 [0.36–14.06] 5.56 [0.48–65.01] 3.01 [0.06–155.01] 4.77 [0.97–23.41]
Education No schooling Elementary Middle School University	0.52 [0.25–1.07] Ref 0.70 [0.37–1.34] 0.66 [0.34–1.29]	1.21 [0.49–2.99] Ref 0.90 [0.45–1.83] 1.12 [0.48–2.62]	1.05 [0.28–3.95] Ref 1.47 [0.48–4.57] 1.24 [0.38–4.06]	-- Ref -- --
Monthly Income No more than $200 $201 to $300 $301 to 500 $501 to $1000 >$1000	Ref 0.97 [0.60–1.56] 0.66 [0.42–1.04] 0.28 [0.16–0.48] 0.38 [0.11–1.30]	Ref 1.04 [0.62–1.74] 0.65 [0.40–1.06] 0.27 [0.15–0.48] 0.36 [0.11–1.18]	Ref 0.76 [0.32–1.79] 1.84 [0.76–4.42] 0.88 [0.32–2.47] 0.16 [0.02–1.11]	Ref 0.54 [0.22–1.28] 1.45 [0.51–4.10] 1.09 [0.37–3.19] 0.16 [0.01–2.05]
Length of Employment Less than 1 year ≤5 Years 6 to 10 years 11 to 15 years ≥ 16 years	Ref 1.08 [0.64–1.82] 1.53 [0.88–2.66] 1.31 [0.68–2.52] 1.12 [0.65–1.94]	Ref 1.18 [0.70–2.00] 1.45 [0.80–2.60] 1.18 [0.59–2.35] 0.76 [0.42–1.38]	Ref 0.92 [0.32–2.61] 1.39 [0.46–4.24] 1.18 [0.33–4.28] 1.04 [0.35–3.10]	Ref -- -- -- --
Zone Urban Rural	Ref 1.44 [1.00–2.05]	Ref 1.29 [0.85–1.97]	Ref 1.08 [0.55–2.13]	Ref --
Sector Primary Secondary Tertiary	Ref 0.82 [0.51–1.33] 0.63 [0.43–0.93]	Ref 1.10 [0.60–2.04] 0.93 [0.54–1.60]	Ref 1.00 [0.40–2.48] 0.54 [0.26–1.12]	Ref 1.15 [0.41–3.22] 0.49 [0.19–1.26]
High Physical Demands of Work No Yes	Ref 1.81 [1.26–2.58]	Ref 1.44 [0.95–2.18]	Ref 2.23 [1.13–4.40]	Ref 2.45 [1.18–5.09]
Exposure to Heat No Yes	Ref 1.56 [1.01–2.41]	Ref 1.16 [0.74–1.83]	Ref 1.36 [0.60–3.08]	Ref 1.37 [0.57–3.30]
Exposure to Humidity No Yes	Ref 2.07 [1.06–4.06]	Ref 2.08 [1.07–4.06]	Ref 2.95 [0.74–11.79]	Ref 2.31 [0.57–9.37]
High Thirst No Yes	Ref 2.11 [1.46–3.05]	Ref 1.93 [1.05–3.57]	Ref 1.48 [0.74–2.99]	Ref 0.48 [0.08–2.69]
No Water Intake No Yes	Ref 1.53 [0.88–2.64]	Ref 1.89 [1.25–2.86]	Ref 0.40 [0.13–1.22]	Ref 1.25 [0.60–2.63]
Use of Analgesics No Yes	Ref 0.51 [0.34–0.75]	Ref 0.51 [0.34–0.76]	Ref 0.57 [0.27–1.20]	Ref 0.86 [0.40–1.85]
High Exposure to Agrochemicals No Yes	Ref 1.33 [0.80–2.23]	Ref --	Ref 1.81 [0.67–4.85]	Ref 1.83 [0.88–3.81]

Hosmer-Lemeshow goodness-of-fit (CKD), *p* = 0.36; Hosmer-Lemeshow goodness-of-fit (work-related CKD), *p* = 0.26; “--”Removed from the selection procedure.

## Data Availability

The datasets generated during this study are available from the corresponding author on reasonable request.

## Abbreviations

II ECCTS	Second Central American Survey of Working Conditions and Health
CKD	chronic kidney disease
CKDu	chronic kidney disease of unknown origin
eGFR	estimated glomerular filtration rate
GIS	geographic information system
DEGREE	Disadvantaged Populations Estimated Glomerular Filtration Rate (eGFR) Epidemiology Study
